# Appropriate Tourniquet Types in the Pediatric Population: A Systematic Review

**DOI:** 10.7759/cureus.14474

**Published:** 2021-04-13

**Authors:** Nathan P Charlton, Craig A Goolsby, David A Zideman, Ian K Maconochie, Peter T Morley, Eunice M Singletary

**Affiliations:** 1 Emergency Medicine, University of Virginia, Charlottesville, USA; 2 Military and Emergency Medicine, Uniformed Services University of the Health Sciences, Bethesda, USA; 3 Pre-Hospital Emergency Medicine, Thames Valley Air Ambulance, Oxford, GBR; 4 Pediatric Emergency Medicine, Imperial College London, London, GBR; 5 Intensive Care Unit, The Royal Melbourne Hospital, Melborne, AUS

**Keywords:** first-aid, tourniquet, pediatric, bleeding, hemorrhage

## Abstract

Trauma is the leading cause of mortality in those aged 1-19, with hemorrhage accounting for up to 40% of all trauma deaths. Manufactured tourniquets are recommended for the control of life-threatening extremity hemorrhage in adults but their use in the pediatric population requires further investigation. We performed a systematic review to evaluate the most appropriate tourniquet design for use in the pediatric population. A literature search of Embase and the Cochran databases of trials and systematic reviews on October 1, 2020 identified 454 unique references, of which 15 were included for full-text screening. Two single-arm observational studies with a high risk of bias evaluated the use of windlass tourniquets in the pediatric population (73 patients, age 2-16 years). The certainty of the evidence was very low. In both studies, conducted on uninjured extremities, the use of a manufactured windlass tourniquet, specifically the Combat Application Tourniquet (C-A-T®) Generation 7, led to the cessation of Doppler detected pulses in 71/71 (100%) of upper extremities and 69/73 (94.5%) of lower extremities. Of the four failures, one participant withdrew due to pain and three tourniquet applications failed to occlude pulses after three turns of the windlass. No controls were used for comparison. In conclusion, two observational studies demonstrated that windlass tourniquets were able to abolish distal pulses in children as young as two years of age and with a minimum limb circumference of 13 cm. These preliminary findings may be helpful for organizations in the creation of guidelines for the management of life-threatening extremity bleeding in children.

## Introduction and background

Trauma is the leading cause of mortality in those aged 1-19 years old [1]. Hemorrhage and hemorrhagic shock account for up to 40% of trauma deaths in the general population [2-4]. In the prehospital period, when injuries may be more amenable to interventions that control bleeding, hemorrhage contributes to mortality in 33% to 56% of all trauma cases, and in one small study, was the most common cause of death prior to the arrival of emergency medical services [3,4]. Manufactured tourniquets have recently been recommended for the control of life-threatening extremity bleeding in adults, but their use in the pediatric population requires further investigation [5-7].

Current tourniquet styles have been tested and used in the military, with most of these tests carried out on individuals ≥18 years of age. Appropriate tourniquet function relies on their ability to be tightened and to apply adequate circumferential pressure to halt distal blood flow. Pediatric limb circumferences are typically much less than those of adults, raising the concern that currently available manufactured tourniquets may not apply adequate circumferential pressure to stop bleeding in smaller children [6]. Some tourniquets, for example, employ a rigid mechanical advantage system (e.g., windlass or ratchet) that precludes their ability to fit circumferences that are smaller than that mechanism.

While limited evidence is available from the civilian population, military data suggests that manufactured tourniquets may also be useful for the treatment of life-threatening extremity hemorrhage in the pediatric population [8,9]. This Systematic Review aims to identify and evaluate the available literature to guide recommendations by the First Aid and Pediatric Life Support Task Forces of the International Liaison Committee on Resuscitation for the use of tourniquets in children with life-threatening extremity hemorrhage.

## Review

We performed a systematic review according to the Cochrane Handbook for Systematic Reviews of Interventions (http://handbook-5-1.cochrane.org) and the results are reported according to the Preferred Reporting Items for Systematic Reviews and Meta-Analyses (PRISMA) guidelines. Our review was registered in PROSPERO (CRD42021229767).

Search strategy

The search strategy was developed in collaboration with a medical librarian (JR) to identify studies that evaluated interventions for the management of severe, life-threatening external bleeding (Appendix Table 2). Search strings were developed for Embase (OVID interface), the Cochrane Central Register of Controlled Trials, and the Cochrane Database of Systematic Reviews. These databases were searched from their inception date until October 1, 2020. For the purpose of this review, the pediatric age of 18 years and younger was chosen by the First Aid and Pediatrics Task Forces and is the same age definition as used in a previous scoping review by the International Liaison Committee on Resuscitation (ILCOR) [6].

Selection criteria

We used the following a priori criteria:

*Population***: **In children (<18 years of age) with severe, life-threatening bleeding from an extremity wound.

*Intervention***:** Commercial elastic wrap tourniquet or commercial ratcheting tourniquet.

*Comparator***: ** Commercial windlass-type tourniquet.

*Outcomes***:** Primary outcomes: mortality, control of bleeding (Critical), blood loss, shock/hypotension, adverse events (Important).

*Study Designs***: ** Randomized controlled trials (RCTs) and non-randomized studies (non-randomized controlled trials, interrupted time series, controlled before-and-after studies, cohort studies) and case series were eligible for inclusion. Unpublished studies (e.g., conference abstracts, trial protocols), modeling studies, animal studies, studies of tourniquets applied solely to maintain a bloodless surgical field, or those relating only to education were excluded. All years and all languages were included as long as there was an English abstract.

Study selection

Two trained reviewers (NC, CG), both experts in prehospital and emergency medicine, independently screened titles and abstracts of the identified citations using the selection criteria. Subsequently, the two reviewers (NC, CG) screened the full text of potentially eligible studies. Disagreements were resolved through discussion and consensus.

Data extraction

One reviewer extracted data into a Microsoft Excel v16 (Microsoft Corporation, Redmond, WA, USA) spreadsheet, which was checked by a second reviewer (NC, CG). Where possible, missing values (e.g., standard deviation) were calculated from the available data (p-values, t-values, confidence intervals, or standard errors).

Risk of bias and certainty of evidence assessment

We summarized the available evidence for each comparison of interest in Grading of Recommendations, Assessment, Development, and Evaluations (GRADE) evidence profile tables (Appendix Table 3). We assessed the certainty of the evidence for each comparison and outcome using the GRADE methodology. We assessed the risk of bias using the ROBINS-I scale for the comparative non-randomized studies. Single-arm studies (without a comparison group) were classified as critical risk of bias.

Data analysis

We used GRADEPro software (Guideline Development Tool [Software], McMaster University, gradepro.org) to create Evidence Profiles (EPs) for reporting a summary of findings and certainty of the evidence per comparison and outcome. We extracted demographics for age (with a minimum and maximum) and for limb circumference when available. For single-arm studies, we reported results as published (raw data and proportions).

Results

Results of the Search

**Figure 1:**
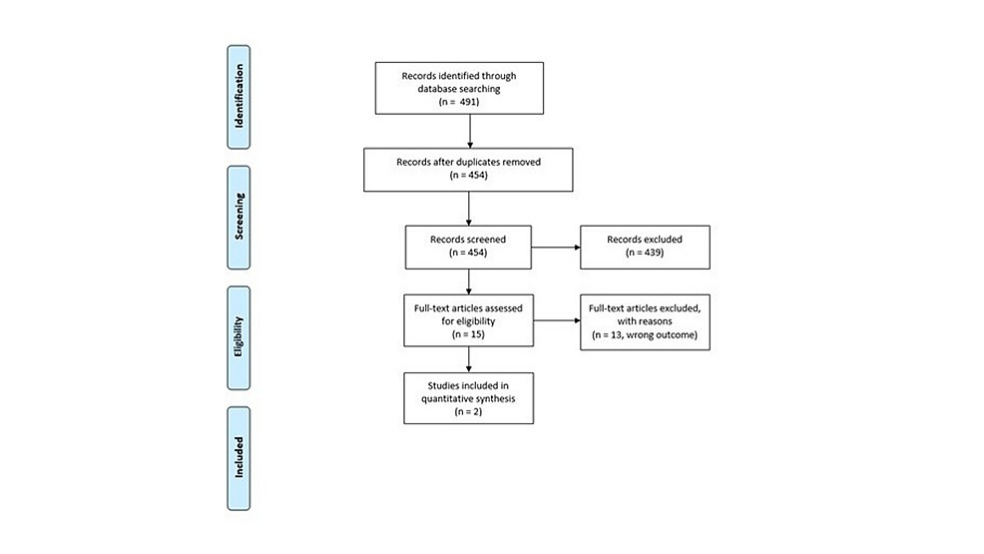
Flow Diagram for the Pediatric Tourniquet Systematic Review

We identified 454 unique references, of which 15 were included for full-text screening. A total of two observational studies (73 patients, age 2-16 years) met the eligibility criteria and were included for analysis; no randomized trials were found (Figure 1).

Study Characteristics

Study characteristics are provided in Table 1. Two single-arm observational studies evaluated the use of windlass tourniquets in the pediatric population (age 2-16 years). These studies had a critical risk of bias (Table 1) and overall, the certainty of the evidence was very low. Certainty downgrades were due to the risk of bias, indirectness, and imprecision. We attempted to assess publication bias, but there were not enough studies for any comparison to allow for a formal evaluation. GRADE evidence profile tables can be found in Appendix Table 3. 

**Table 1:** Characteristics of Included Studies for the Pediatric Tourniquet Systematic Review CAT: Combat Application Tourniquet

Study	Study design	Intervention	Comparison	Participants	Setting	Key Results	Risk of bias (ROBINS-I)
Harke 2019 [10]	Observational	Combat Application Tourniquets (C-A-T®) (120 applications)	No comparison	Sixty participants 6-16 years of age were recruited as a convenience sample from an orthopedic clinic and had a CAT Generation 7 applied to one upper extremity at the mid-biceps level and one lower extremity at the mid-thigh level. A non-injured extremity was used.	Outpatient orthopedic clinic. All tourniquets were applied by the researchers and no apparent blinding occurred. Successful application was determined by cessation of the distal pulse by Doppler with a maximum of three windlass turns to limit pain.	The CAT was successful in occluding arterial flow in 100% (60/60) of upper extremities and 93% (56/60) lower extremities. Distal pulses occlusion occurred in both upper and lower extremities of children age six and over with a limb circumference ≥16 cm. Three applications in the lower extremity failed to occlude pulses after three windlass turns and on one application a participant withdrew due to pain.	Critical
Kelly 2020 [11]	Observational	Combat Application Tourniquets (C-A-T®) (24 applications)	No comparison	Thirteen participant age two to seven years. A non-injured extremity was used. Successful application was determined by cessation of the distal pulse by Doppler.	Children undergoing elective orthopedic surgery while under anesthesia. All tourniquets were applied by the researchers and no apparent blinding occurred. The outcome was cessation of distal pulse as detected by doppler.	Tourniquets were placed on 24 limbs (11 upper extremities and 13 lower extremities) with a 100% success rate in occluding distal pulses. The minimal limb circumference tested was 13 cm in a two year-old child.	Critical

Study Findings

Results are reported with a focus on those outcomes which are felt to be the most important for guideline development. Study characteristics and results can be found in Table 1 and in Appendix Table 3.

Cessation of Bleeding

For the critical outcome of bleeding cessation, no studies were found that compared one tourniquet type with another tourniquet type in children <18 years of age. We found two observational studies with very low certainty evidence (downgraded for risk of bias, indirectness, and imprecision) enrolling a total of 73 children, age 2-16 years, that used distal pulse cessation, as detected by Doppler ultrasound, as the outcome and surrogate for the control of bleeding [10,11].

One observational study published in 2019 by Harke et. al. [10] evaluated tourniquet use in 60 children age 6-16 years who visited an orthopedic office. Investigators applied a Combat Application Tourniquet (C-A-T®) Generation 7 to one uninjured upper extremity at the mid-biceps level and one uninjured lower extremity at the mid-thigh level. A successful application was determined by distal pulse cessation, as detected by Doppler ultrasound, as the surrogate outcome for the control of bleeding. For a successful application, a maximum of three windlass turns (a total of 1080°) were selected a priori to limit pain. The C-A-T® was successful in occluding distal pulses in 100% (60/60) of upper extremities and 93% (56/60) of lower extremities. One participant stopped lower extremity tourniquet application before pulse cessation due to excess pain, and three tourniquet applications failed to occlude pulses after a protocol limit of three turns of the windlass. No controls were used for comparison. The C-A-T® Gen 7 windlass tourniquet was successful in occluding distal pulses in both upper and lower extremities of those children aged 6 years and over with a limb circumference ≥16 cm.

In 2020 Kelly et. al. [11] published an observational study evaluating tourniquet use in 13 children aged 2-7 years. In this study, patients undergoing elective orthopedic surgery who were anesthetized and being prepared for surgery in an operating room had a C-A-T® Gen 7 tourniquet placed on one or more uninjured extremities. All tourniquets were placed at the most proximal limb position possible by the researchers. A successful application was determined by distal pulse cessation, as detected by Doppler ultrasound, as the surrogate outcome for the control of bleeding. Tourniquets were placed on 24 limbs (11 upper extremities and 13 lower extremities) with a 100% success rate in occluding distal pulses. The minimal limb circumference tested was 13 cm in a 2-year-old child. No controls were used for comparison. 

Adverse Events

For the important outcome of adverse events, no studies were found that compared one tourniquet type with another tourniquet type in children <18 years of age. We found one observational study [10] with very low certainty evidence (downgraded for bias, indirectness, and imprecision) enrolling a total of 60 children aged 6-16 years, in which a windlass tourniquet was used to evaluate cessation of distal pulses in uninjured extremities. In this study, of the 120 applications of the tourniquet to upper (n=60) and lower (n=60) extremities, only one tourniquet application (1/120) caused a child (unknown age) to experience enough pain during tightening of the tourniquet to halt its application. No other adverse events were reported.

Discussion

Our systematic review identified two studies reporting outcomes of interest for the use of tourniquets to control life-threatening bleeding in the pediatric population. While high-quality studies comparing tourniquet application for the control of bleeding in the pediatric population are generally lacking, our systematic review summarizes the best available evidence for the creation of guidelines for immediate responders. In the evaluated studies, a windlass tourniquet resulted in pulse cessation in 140/144 (97%) applications: 71/71 (100%) in upper extremity and 69/73 (94.5%) in lower extremity applications [10,11]. This evidence is indirect, with both studies performed in a medical setting, using the same type of windlass tourniquet and using the loss of a Doppler detected pulse as an indication of a cessation of bleeding. Based on this evidence, a windlass-style tourniquet will abolish distal pulses in children down to two years of age, with a minimal limb circumference of 13 cm [10,11]. Evidence from human studies for other tourniquet types was not found in this review.

Both the Pediatric Trauma Society 2017 position statement and the Pediatric Working Group for the 2013 Committee for Tactical Emergency Casualty Care recommend tourniquet use in the prehospital care of pediatric trauma patients [12,13]. The Pediatric Trauma Society recommends the use of tourniquets in children with exsanguinating extremity hemorrhage in the prehospital setting and during resuscitation of children if direct pressure fails to control the hemorrhage or if attempting direct pressure is too resource intensive [13]. The Committee for Tactical Emergency Casualty Care Pediatric Working Group recommends tourniquets for life-threatening extremity hemorrhage in children as first-line therapy in both direct threat care (care under fire) and indirect threat care [12].

In this review we found a lack of direct comparative evidence for the best type of tourniquet in infants and children, highlighting an urgent need for future research. Successful tourniquet application depends on the ability to provide circumferential pressure on the extremity to stop distal blood flow. This typically involves applying a strap and then tightening with a mechanism that provides a mechanical advantage. Whilst studies have demonstrated that failure to initially tighten the strap often leads to tourniquet failure in adults, in the pediatric population, the mechanism of applying mechanical advantage may, in some cases, be too large and rigid to allow for adequate tightening [14,15]. In the current review, the C-A-T® was able to occlude distal arterial blood flow in both the upper and lower extremities in children as young as two years of age with a minimum limb circumference of 13 cm. However, other brands of windlass rod tourniquets may vary in their ability to successfully tighten around small limb diameters.

Use of other tightening mechanisms, such as ratcheting tourniquets, has been suggested for use in children, but the evidence is lacking. No evidence from human studies was available for other tourniquet mechanisms, however, indirect evidence is available from three experimental studies that evaluate tourniquets in models representing a small limb diameter [16-18]. While these studies evaluate a broader range of tourniquet mechanisms, including ratcheting and elastic wrap, the experiments were conducted on manikins and in models using PVC pipe and stair rails and were excluded by our initial search strategy. A review of the indirect evidence from these studies suggests that the rigid mechanisms of some tourniquets can preclude successful application in small limb diameters.

We were unable to identify any complications of the use of a tourniquet in this review. The only reported adverse effect was pain in one tourniquet application in a single study [10]. While pain led to tourniquet failure in this application, this result is understandable as it would have been unethical to inflict pain in a volunteer study in a pediatric population [10]. Pain upon tourniquet placement is consistent with studies of adult tourniquet use and is an expected consequence of tourniquet application [7].

There is no human evidence for the use of either manufactured or improvised tourniquets in children less than two years of age. However, for this age group, the smaller body size and relatively lower blood pressure would possibly make the application of direct manual pressure to control bleeding more efficacious than in adults [19]. Based on adult literature, the use of a hemostatic dressing, if available would likely be beneficial as an adjunctive therapy to direct manual pressure for the control of life-threatening extremity bleeding [7].

Limitations

There are a number of limitations acknowledged with this review. First and foremost, only two observational human studies were identified in the pediatric population. Both studies used the same C-A-T® tourniquet in healthy volunteers. This greatly limits the ability to recommend the most appropriate tourniquet types for use in the pediatric population. In addition, researchers applied all tourniquets and the outcome of cessation of distal pulses by Doppler was used as a surrogate outcome for the cessation of bleeding. How these factors translate to tourniquet use in children with life-threatening hemorrhage applied by lay or prehospital providers remains unknown. Lastly, the youngest study participant was age two and had a limb circumference of 13 cm. It is not known whether tourniquets may be useful for infants or children with a smaller limb circumference. More human studies are needed to determine whether other tourniquet types can be used successfully in the pediatric population and to define the lower age limits at which these tourniquets can be successfully applied to either the upper or lower extremities.

## Conclusions

In two observational studies of tourniquet use to control life-threatening bleeding in the pediatric population, a manufactured windlass tourniquet, specifically the Combat Application Tourniquet® Generation 7, was able to successfully abolish distal pulses in children as young as two years of age and with a minimum limb circumference of 13 cm. While some indirect evidence is available, there is no direct human evidence for other tourniquet designs in children. These preliminary findings may be helpful for organizations in the creation of guidelines for pediatric life-threatening extremity bleeding.

## Appendices

**Table 2:** Search Strategy for the Pediatric Tourniquet Systematic Review

Embase Conducted October 1, 2020		
No.	Query	Results
#1	'tourniquet'/exp OR tourniquet$:ti,ab,kw,de OR windlass:ti,ab,kw,de OR ((elastic NEAR/3 (ring OR band OR wrap OR strap)):ti,ab,kw,de)	11,243
#2	#1 NOT ('snakebite'/de OR 'spider bite'/de OR 'venom'/de OR 'hypospadias'/de OR 'arthroscopy'/exp OR snake:kw,de OR spider:kw,de OR hypospadias:kw,de OR arthroscop*:kw,de)	10,619
#3	#2 NOT ([conference abstract]/lim OR [conference review]/lim OR [editorial]/lim OR [erratum]/lim OR [letter]/lim OR [note]/lim OR [book]/lim OR 'case report'/de)	7,652
#4	'bleeding'/de OR 'wound hemmorhage' OR haemorrhag*:ti,ab OR hemorrhag*:ti,ab OR exsanguinat*:ti,ab OR bleed*:ti,ab OR 'blood loss':ti,ab,kw,de	791,116
#5	'traumatic amputation'/de OR trauma*:ti,ab OR amputat*:ti,ab	530,698
#6	'arm injury'/de OR 'leg injury'/de OR 'limb injury'/de	18,791
#7	'battle injury'/de OR 'blast injury'/de	9,003
#8	#3 AND #4	1,652
#9	#3 AND #5	754
#10	#3 AND #6	128
#11	#3 AND #7	134
#12	#8 OR #9 OR #10 OR #11	2,099
#13	'newborn'/exp OR 'infant'/exp OR 'child'/exp OR 'adolescent'/exp OR 'pediatrics'/exp OR infant$:de,kw,ab,ti OR baby:de,kw,ab,ti OR babies:de,kw,ab,ti OR paediatric$:de,kw,ab,ti OR pediatric$:de,kw,ab,ti OR kid:de,kw,ab,ti OR kids:de,kw,ab,ti OR child*:de,kw,ab,ti OR 'pre-adolescen*':de,kw,ab,ti OR 'preadolescen*':de,kw,ab,ti OR adolescen*:de,kw,ab,ti OR teenager$:de,kw,ab,ti OR juvenile$:de,kw,ab,ti OR youth$:de,kw,ab,ti OR ((young NEAR/3 (person$ OR people)):de,kw,ab,ti)	4,632,610
#14	#12 AND #13	250
#15	tourniquet:ti OR tourniquet$:ti OR windlass:ti OR ((elastic NEAR/3 (ring OR band OR wrap OR strap)):ti)	3,117
#16	#15 NOT ('snakebite'/de OR 'spider bite'/de OR 'venom'/de OR 'hypospadias'/de OR 'arthroscopy'/exp OR snake:kw,de OR spider:kw,de OR hypospadias:kw,de OR arthroscop*:kw,de)	3,020
#17	#16 NOT ([conference abstract]/lim OR [conference review]/lim OR [editorial]/lim OR [erratum]/lim OR [letter]/lim OR [note]/lim OR [book]/lim OR 'case report'/de)	2,275
#18	#13 AND #17	171
#19	#14 OR #18	355

Cochrane Central Register of Controlled Trials and the Cochrane Database of Systematic Reviews Conducted October 1, 2020		
No.	Search	Hits
#1	(tourniquet* or windlass*):ti,ab,kw or [mh tourniquets]	1,991
#2	elastic near/3 (band* or strap* or wrap* or ring*):ti,ab,kw	811
#3	#1 or #2	2,790
#4	newborn OR infant* OR child* OR adolescent* OR pediatric* OR paediatric* OR baby:kw,ab,ti OR babies:kw,ab,ti OR "pre-adolescen*":kw,ab,ti OR 'preadolescen*':kw,ab,ti OR teenager$:kw,ab,ti OR juvenile*:kw,ab,ti OR youth*:kw,ab,ti OR ((young NEAR/3 (person* OR people)):kw,ab,ti) OR [mh child] OR [mh adolescent] OR [mh infant]	298,563
#5	#3 AND #4 in Cochrane Reviews, Cochrane Protocols	17
#6	(tourniquet* or windlass*):ti or [mh tourniquets] OR elastic near/3 (band* or strap* or wrap* or ring*):ti	1104
#7	#6 AND #4	121
#8	#7 in Trials	119

**Table 3:** Evidence Profile for the Pediatric Tourniquet Systematic Review CI: Confidence interval ^a ^researchers applied tourniquets ^b^ no apparent blinding ^c^ cessation of pulses used as a surrogate for bleeding cessation ^d^ low number of participants ^1^  Harcke et al. [10] ^2^ Kelly et al. [11]

Question: Should a windlass tourniquet compared with no tourniquet be used for life-threatening extremity bleeding in children?												
Certainty assessment							№ of patients		Effect		Certainty	Importance
№ of studies	Study design	Risk of bias	Inconsistency	Indirectness	Imprecision	Other considerations	Windlass tourniquet	no tourniquet	Relative (95% CI)	Absolute (95% CI)		
Cessation of bleeding in upper extremities (assessed with: Occlusion of distal pulses in upper extremity by Doppler)												
2 ^1,2^	observational studies	serious ^a,b^	not serious	serious ^c^	serious ^d^	none	71/71 (100.0%)	0/0	not estimable		⨁◯◯◯ VERY LOW	CRITICAL
Cessation of bleeding in lower extremities (assessed with: Occlusion of distal pulses in lower extremity by Doppler)												
2 ^1,2^	observational studies	serious ^a,b^	not serious	serious ^c^	serious ^d^	none	69/73 (94.5%)	0/0	not estimable		⨁◯◯◯ VERY LOW	CRITICAL
Adverse events												
1 ^1^	observational studies	serious ^a,b^	not serious	serious ^c^	serious ^d^	none	1/120 (0.8%)	0/0	not estimable		⨁◯◯◯ VERY LOW	IMPORTANT
